# Cyanocobalamin Ultraflexible Lipid Vesicles: Characterization and In Vitro Evaluation of Drug-Skin Depth Profiles

**DOI:** 10.3390/pharmaceutics13030418

**Published:** 2021-03-20

**Authors:** Antonio José Guillot, Enrique Jornet-Mollá, Natalia Landsberg, Carmen Milián-Guimerá, M. Carmen Montesinos, Teresa M. Garrigues, Ana Melero

**Affiliations:** 1 Department of Pharmacy and Pharmaceutical Technology and Parasitology, Faculty of Pharmacy, University of Valencia, Avda. Vicent Andrés Estellés s/n, 46100 Burjassot, Spain; antonio.guillot@uv.es (A.J.G.); enjormo@alumni.uv.es (E.J.-M.); landsbergnat95@gmail.com (N.L.); carmenmilianguimera@gmail.com (C.M.-G.); ana.melero@uv.es (A.M.); 2 Department of Pharmacology, Faculty of Pharmacy, University of Valencia, Avda. Vicent Andrés Estellés s/n, 46100 Burjassot, Spain; 3 Center of Molecular Recognition and Technological Development (IDM), Polytechnic University of Valencia and University of Valencia, Avda. Vicent Andrés Estellés s/n, 46100 Burjassot, Spain

**Keywords:** cyanocobalamin, vitamin B12, atopic dermatitis, psoriasis, liposomes, transferosomes, lipid vesicles, skin topical delivery

## Abstract

Atopic dermatitis (AD) and psoriasis are the most common chronic inflammatory skin disorders, which importantly affect the quality of life of patients who suffer them. Among other causes, nitric oxide has been reported as part of the triggering factors in the pathogenesis of both conditions. Cyanocobalamin (vitamin B_12_) has shown efficacy as a nitric oxide scavenger and some clinical trials have given positive outcomes in its use for treating skin pathologies. Passive skin diffusion is possible only for drugs with low molecular weights and intermediate lipophilicity. Unfortunately, the molecular weight and hydrophilicity of vitamin B_12_ do not predict its effective diffusion through the skin. The aim of this work was to design new lipid vesicles to encapsulate the vitamin B_12_ to enhance its skin penetration. Nine prototypes of vesicles were generated and characterized in terms of size, polydispersity, surface charge, drug encapsulation, flexibility, and stability with positive results. Additionally, their ability to release the drug content in a controlled manner was demonstrated. Finally, we found that these lipid vesicle formulations facilitated the penetration of cyanocobalamin to the deeper layers of the skin. The present work shows a promising system to effectively administer vitamin B_12_ topically, which could be of interest in the treatment of skin diseases such as AD and psoriasis.

## 1. Introduction

Eczema or atopic dermatitis (AD) and psoriasis are the most common chronic inflammatory skin diseases. AD is characterized by pruritus, dry skin, eczematous injuries, and lichenification [1]. The onset of AD commonly occurs during childhood, and it is associated with a significant morbidity and reduced quality of life. The prevalence of AD has increased over the past 30 years, and its incidence has increased 2- to 3-fold in recent years. Approximately, 20% of children and 1–3% of adults are affected by this disorder [2]. Although the pathogenesis of AD is not completely understood, it has been associated with various factors: alteration of the skin barrier function, immune dysregulation, infections, and environmental processes. The alterations in the skin barrier function may be caused by deficient levels of ceramides and proteins involved in keratinocyte differentiation and prevention of transepidermal water loss [3,4]. Several genetic factors, such as mutations in filaggrin genes, could cause this situation [5]. The defective barrier function allows the penetration of allergens and irritant agents that trigger inflammation through Th2 responses (with increased interleukins: IL-4, IL-5, IL-13 cytokines) in the acute phase and Th1 responses (with increased Interferon (IFN)-gamma and IL-12) in chronic injuries [6]. In addition, the usual scratching stimulates keratinocytes to release other inflammatory cytokines like Tumoral Necrosis Factor (TNF)-alpha, IL-1, and IL-6, which perpetuate chronic inflammation. Ultimately, the modified state of the skin contributes to bacteria colonization, which further worsens the disorder [6].

Likewise, psoriasis is a chronic and inflammatory skin disease with a marked immune component. Its most typical skin manifestation consists in erythematous plaques, often spread over large areas of the body. The worldwide prevalence varies from 1% to 8%, depending on the geographical areas. Although it can begin at any age, its onset frequently occurs in adulthood, with one out of three cases during childhood [7]. A major characteristic in psoriasis pathogenesis is the inappropriate activation of the immune system, which leads to keratinocyte hyperplasia, altered T cell function and angiogenesis, among others [8,9,10]. Besides, oxidative stress and increased expression of insulin-like growth factor-1 (IGF-1) and epidermal growth factor (EGF) also participate in the pathogenesis [11].

The main goals of the treatment of these pathologies include restoring skin barrier, limiting itching, decreasing inflammation, and controlling immune alterations. To achieve them, a wide plethora of drugs may be used, with systemic immunosuppressive agents, topical corticosteroids, and topical calcineurin inhibitors (TCIs) being the first-line drugs in the pharmacological management of AD and psoriasis [12]. However, systemic treatments are not exempt of potential serious adverse side effects, such as kidney and liver disfunction or myelosuppression, which complicate the management of severe cases [13,14,15]. Local side effects associated with long-term use of topical steroids and TCIs are relatively common, such as skin atrophy, ecchymosis, erosions, striae, delayed wound healing, purpura, easy bruising, and acne [16]. In addition, the Food and Drug Administration (FDA) has reported warnings for the use of some TCIs due to the potential risk of skin cancer and lymphomas [17].

The proinflammatory cytokines involved in AD and psoriasis stimulate the expression of inducible nitric oxide synthase (iNOS) in keratinocytes. In consequence, skin lesions of AD and psoriasis present higher levels of nitric oxide, which has been implicated in the pathogenesis of atopic eczema and psoriasis [18]. Oral and topical cyanocobalamin (B12) have been used as an alternative for preventing or ameliorating AD and psoriasis [19]. Although the first trials dated from the 1960s, not many studies have been carried out recently [20]. Januchowski et al. compared the effect of B12 cream (0.07%) with a moisturizer base for the treatment of childhood eczema in a double-blinded randomized trial using the SCORAD scale (Scoring of Atopic Dermatitis). Intraindividual comparisons showed significant differences in reduction of SCORAD values between B12 cream and placebo. Particularly, topical B12 significantly improved treated skin more than placebo at 2 and 4 weeks. Recently, mild-to-moderate plaque psoriasis has been treated with topical B12 ointments for 12 weeks in two clinical randomized trials [21]. Strücker et al. compared the effect of B12 with calcipotriol (vitamin D_3_ analogue), obtaining similar decreases in the PASI score (Psoriasis Area Severity Index) after the studied period. Moreover, there was a marked diminution of the efficacy of calcipotriol after 4 weeks of therapy, whereas the efficacy of B12 remained largely constant over the whole observation period, making it more suitable for long-term use [22]. Similarly, a positive outcome was obtained when comparing B12 and emollient cream in the study done by Del Luca et al. The differences in PASI reduction were noticeable at week 2 and increased during the following 10 weeks. Two weeks after the end of the treatment, the psoriatic lesions evolved negatively (especially the ones treated with B12), proving the potential role of cyanocobalamin-based formulation in the treatment of epidermal disorders [23].

Despite these promising results, the molecular weight (1355 Da) and hydrophilicity of B12 limit its diffusion through intact human skin, given that one of the main functions of the skin is to prevent the penetration of exogenous substances to the body [24]. As the barrier function is recovered during treatment, B12 permeability might be gradually reduced. Research on cutaneous permeability has shown that skin diffusion may be improved by using a suitable formulation, and it can even be used for systemic administration [25]. The strategies to overcome the limitations for drug permeation through the skin are addressed to disrupt the barrier function of the stratum corneum or to change the drug physicochemical properties. In this sense, although water-based vehicles have been reported to be suitable for B12 topical delivery, other studies suggest that the use of chemical enhancers, such as ethanolic solutions and oleic acid/propyleneglycol-based formulations, provide a better diffusion through the skin [26,27]. In addition, other active strategies, such as iontophoresis and microneedeling, showed higher penetration rates for B12 as well [27,28].

Nanosystems are another strategy to enhance drug delivery through the skin, consisting of the design of different types of nanocarriers, such as lipid nanoparticles, polymeric micelles, and poly (β-amino ester) [29,30,31,32,33,34]. Among those, liposomal-type systems have shown promising results in transdermal purposes [35,36]. Liposomes are microscopic vesicles with an aqueous core surrounded by one or more outer layers composed of non-toxic and biodegradable phospholipids, which are commonly used for their ability to encapsulate hydrophilic and lipophilic drugs [37]. Biomedical research has led to the development of modified lipid vesicles that include surfactants (transfersomes) or ethanol (ethosomes) in their composition, which provide greater flexibility, causing different carrier–skin interactions and thus enhancing skin drug delivery [38,39]. Cyanocobalamin liposomes have already been prepared by Arsalan et al. to improve its photostability and by Vitetta et al. to achieve systemic absorption after oral administration [40,41].

Based on these criteria, the aim of this study was to prepare and characterize stable lipid vesicles—liposomes, transfersomes, and ethosomes—to improve cyanocobalamin skin penetration.

## 2. Materials and Methods

### 2.1. Materials

Phospholipon 90G (soybean phosphatidylcholine) (P90G) was kindly donated by Lipoid (Steinhausen, Switzerland). Cholesterol and B12 (purity 95%) were purchased from Acofarma (Madrid, Spain). Tween 80, phosphate salts, and sodium chloride to prepare buffers were from Scharlab (Sentmenat, Spain). Regenerated cellulose dialysis membranes with a cut-off of 14kDa (Spectra/Por^®^ molecular porous membrane tubing) for the in vitro release studies were acquired from Repligen (Waltham, MA, USA). HPLC-quality water obtained by Milli-Q purification (Millipore, Madrid, Spain) with resistance > 18 MΩ cm and TOC < 10 ppb was used for liposome reconstitution and HPLC analysis. Methanol for the HPLC mobile phase was purchased at VWR International (Radnor, PA, USA). For the tape-stripping studies, adhesive tape Tesafilm kristall-klar (33 mm × 19 mm, cut to 30 mm × 19 mm sections) was obtained from Tesa (Hamburg, Germany); Parafilm Sealing Film—50 mm (2”) Film Width × Roll Length 75 m (Parafilm, Madrid, Spain) was used; glass beads were obtained from MERCK (Madrid, Spain). Pig ears were procured from a local slaughterhouse (Mercavalencia, Valencia, Spain).

### 2.2. Cyanocobalamin Quantification. Development and Validation of a HPLC Method

B12 was quantified by HPLC using a PerkinElmer^®^ Series 200 equipped with a UV detector settled at 360nm and a Kromasil^®^ C18 HPLC column of 5 μm particle size, pore size 100 Å, L × I.D. 150 mm × 4.6 mm. The volume injection was 50 µL, and the mobile phase consisted of a isocratic mixture of methanol:water (30:70), delivered at a flow rate of 1 mL min^−1^. The B12 retention time was 3.6 min, and the analysis duration was 5 min.

For the validation of the method, a stock standard solution of B12 (100 µg/mL) was prepared by dissolving 0.01 g of the drug in 100 mL of water. Ten working solutions (100, 10, 5, 2.5, 1.25, 0.625, 0.312, 0.156, 0.078, and 0.039 µg/mL) were prepared by diluting an adequate amount of stock standard solution with water. All analyses were performed in triplicate (*n* = 3).

### 2.3. Preparation of Liposomes, Transferosomes and Ethosomes

Several formulations of liposomes (L), transferosomes (T), and ethosomes (E) were prepared by the classic film-hydration method [42,43]. Their quantitative composition, reconstitution conditions, and the methods used to purify them are shown in Table 1. Three batches of each lipid vesicle type were prepared, empty and loaded with a variable amount of B12. Cyanocobalamin was added to the organic phase dispersion or included in the liposomal reconstitution solution (80% *w/v* of the solubility limit to achieve the maximum loading possible). Briefly, P90G, cholesterol (P90G:Chol molar ratio 17:1) or surfactants (15% *w/w* of lipid), and cyanocobalamin (except in the blank formulations and formulations reconstituted with B12 solution) were dissolved in methanol. The solvent was evaporated using a rotary evaporator (BUCHI R-210) under stirring (Heidolph RZR-2021) at 50 °C and 100 mbar. The resulting thin film was hydrated by addition of PBS 7.4 pH or B12 solution and then stirred for 1 h at 50 °C to obtain a multi-lamellar vesicle (MLV) dispersion. Transfersome batches were prepared by the same process with the exception that cholesterol was replaced by Tween 80. The ethosomal batches were prepared by the Touitou method [44], dissolving P90G and B12 in ethanol. Then, an appropriate amount of water was added at 12 ± 0.5 mL/h in a sealed beaker under stirring (710 ± 5 rpm), in a water bath tempered at 30 °C. Concurrently, the option of adding B12 dissolved in the water flow was also explored. The system was kept under stirring for 5 min after the addition of the water.

Once MLV were obtained, their size was reduced by sonication and membrane extrusion. In the case of L and T, they were firstly sonicated at 50 °C for 2 h (Elmasonic S60H), then cooled down to 4 °C and extruded through a 200 μm membrane at 30 °C, using a LiposoFast-Basic Extruder, Avestin (20 times) [45]. Ethosomes were sonicated for 1 h at room temperature and reduced by the same extrusion process. After size reduction, the samples were purified by centrifugation (12,800× *g*, 30 min) or washed in 2 L PBS at 4 °C for 24 h to compare both methods [46,47]. The batches were then stored at 4 °C protected from the light.

### 2.4. Characterization of the Lipid Vesicles

#### 2.4.1. Determination of Entrapment Efficiency

Entrapment efficiency (EE) was determined directly by calculating the amount of B12 encapsulated in lipid vesicle dispersions using the following equation (Equation (1)) [48]:

EE(%) = (Q_e_/Q_t_) ∙ 100 (1)

where Q_e_ is the amount of B12 encapsulated, and Q_t_ is the amount of B12 used for batch preparation.

For this, 0.5 mL of lipid vesicles dispersion was incubated for 1 h with a mixture of water:methanol:sodium doceyl sulfate 1% (45:45:10) to dissolve all the vesicle components [49,50,51]. The final mixture was filtered, and B12 content was analysed by HPLC.

#### 2.4.2. Determination of Particle Size, Polidispersity Index (PDI) and Zeta-Potential

Vesicular size (average diameter), polydispersity index (PDI), and zeta potential were measured by means of a Malvern nanozetasizer (Malvern, UK). Dynamic light scattering (DLS) mode was used to measure the vesicular size and PDI, while laser doppler electrophoresis (LDE) was used to determine the zeta potential. The temperature was set at 25 °C in all cases, and each sample (*n* = 3) was analysed in triplicate [52].

#### 2.4.3. Determination of Phospholipid Content of Lipid Vesicles

The method of Rouser et al. was used to determine the amount of phosphatidylcholine (PC) incorporated into the different vesicles (*n* = 3) [53]. Briefly, 100 µl of the liposomal aqueous samples was heated at 270 °C until complete liquid evaporation, followed by addition of 450 μL of HClO_4_ (70% *v/v*). Next, the mixture was heated to 250 °C for 30 min. After cooling down, 3.5 mL of water, 500 μL of ammonium molybdate (2.5% *w/v*), and 500 μL of ascorbic acid (10% *w/v*) were added. The mixture was vortexed and incubated at 100 °C for 7 min. After the tubes were cooled down, the absorbance was measured at 820 nm (spectrophotometer HITACHI U-2900, Milan, Italy).

#### 2.4.4. Evaluation of Lipid Vesicle Flexibility

The flexibility of the different lipid vesicles was estimated indirectly by extrusion through a 100 nm membrane in cold. For this, 500 µL of each formulation (*n* = 3) was extruded 9 times at room temperature. The final collected volume was also recorded and the relative decrease ratio in particle size was calculated [54].

#### 2.4.5. Stability Studies

The physical stability of the vesicles was checked weekly during 60 days in terms of size and PDI by DLS. The stability of the vesicle suspensions was determined using Turbiscan^TM^ LAB Stability Analyzer (Formulaction SA, L’Union, France) by checking the phenomena of coalescence, flocculation, creaming, sedimentation, and clarification during the 24 h after the suspension was formulated [55]. The chemical stability of the B12 was assessed by determination of drug content every week for 3 months.

### 2.5. Release Studies

In vitro release studies were carried out using static Franz-type diffusion cells with an effective diffusion area of 1.76 cm^2^. A 500 µL of each formulation was added to the donor chamber, and the receptor chamber was filled with 12 mL of PBS (pH 7.4) (*n* = 6). Temperature was maintained at 32 °C throughout the experiment. A Spectra/Por^®^ molecular porous membrane was used to separate donor and acceptor compartments. Both the donor compartment and the sampling port were covered with Parafilm^®^ to avoid leakage and solvent evaporation. Samples of 400 μL were collected at 1, 2, 3, 4, 5, 6, 7, 8, 9, 10, 24, 48, and 72 h. At every sampling time, the volume was replaced with pre-warmed PBS to guarantee sink conditions [56].

The released B12 was quantified using the same HPLC analytical method described above, and the cumulative amounts of B12 versus time were calculated. The total drug amount released was calculated according to the previous determination of the drug content and plotted versus time. The release profiles were fitted to different mathematical kinetic models: Higuchi, Korsmeyer–Peppas, Kim, Peppas–Sahlin, zero order and first order. For each case, the Akaike information criterion (AIC) was calculated to determine the optimal models to explain the experimental data. The correlation coefficient (*R*^2^) of the most accurate model was reported as an indicator of the proportion of variation of the results that could be explained by the model [57,58].

The Higuchi model assumes that the release process is carried out only by passive diffusion and is governed by the following equation (Equation (2)) [59]:

M_t_/M_∞_ = k ∙ √t (2)

where M_t_ is the amount released at time t, M_∞_ is the maximum amount of drug released, M_t_/M_∞_ is the fraction of the amount of drug released at time t, k is the constant that governs the process.

The Korsmeyer–Peppas model or power-law is described with the following equation (Equation (3)) [60,61]:

M_t_/M_∞_ = k ∙ t^n^ (3)

where M_t_ is the amount released at time t, M_∞_ is the maximum amount of drug released, M_t_/M_∞_ is the fraction of the amount of drug released at time t, k is the constant that governs the process and explains the characteristics of the system, and n is the diffusion release exponent. Values of n < 0.5 are indicative that the release process is carried out by passive diffusion; values of 0.85 < n < 1 show that the process is governed mainly by relaxation, and intermediate values 0.5 < n < 0.85 indicate the existence of both phenomena (anomalous transport).

As a variation of the Korsmeyer–Peppas equation, Kim et al. proposed a modification (Equation (4)) to assess the possible burst effect of the formulation [62]:

M_t_/M_∞_ = k ∙ t^n^ + n (4)

where b is the parameter corresponding to the burst effect.

The Peppas–Sahlin model considers that release may occur through the processes of passive diffusion and relaxation, each represented by a constant (Equation (5)) [63]:

M_t_/M_∞_ = k_1_ ∙ t^n^ + k_2_ ∙ t^n^ (5)

where k_1_ and k_2_ are, respectively, the constants associated with the processes of drug release by passive diffusion and relaxation, and n is the diffusion release exponent.

Representative theoretical models, zero order (Equation (6)) and first order (Equation (7)), are described by the following equations [64]:

M_t_/M_∞_ = k_d_ ∙ t (6)

M_t_/M_∞_ = 1 − e^−kd ∙ t^ (7)

where k_d_ is the constant of diffusion release that governs the process.

### 2.6. Tape-Stripping Studies. Drug Penetration through the Skin and Stratum Corneum Depth

The assay was performed using porcine skin samples. Skin was removed from the connective tissues and placed onto a glass slide, with the outside layer facing upwards. An aluminium mask was placed over it, leaving the application area uncovered. One hundred microliters of each formulation containing lipid vesicles was applied to the delimited application area of skin. The system was then incubated at 32 °C for 2, 4, and 6 h. Each test was performed in triplicate (*n* = 3). After incubation, 20 strips of adhesive tape were applied sequentially to the skin using a roller, according to the standardized procedure in our lab, and then removed with a forceps [65]. The amount of stratum corneum removed was determined by infrared densitometry (SquameScan™ 850A device, Wetzlar, Germany), and strips were grouped in different pools, following the sequence: 1, 2, 3–5, 6–10, 11–15, 16–20 [66]. The B12 amount present in each sample was extracted overnight from the strips using a methanol:water mixture (50:50 *v/v*) as extraction solvent, since it afforded an optimal B12 recovery ratio within the established range (100 ± 20%) [67]. Subsequently, B12 was quantified by HPLC with no interferences with the analytical method.

### 2.7. Data Analysis and Statistical Analysis

All data processing was performed in Microsoft Excel 2016 (Redmond, WA, USA) and SPSS version 22.0 (IBM Corp, Armonk, NY, USA). Data are expressed as the mean ± standard deviation (SD) unless otherwise stated. Statistical differences were determined using one-way ANOVA followed by Tukey’s post hoc analysis for tests with two variables or two-way ANOVA followed by Bonferroni’s post hoc analysis for tests with three variables, where *p*-values below 5% (*p* < 0.05) were considered significant.

## 3. Results and Discussion

### 3.1. Characterization of Lipid Vesicles

During the lipid vesicle design stage, different factors were considered that could potentially influence their properties, based on previous knowledge about this type of nanocarriers. In this sense, two essential factors are the composition and proportion of components, since they can affect important properties, such as the size, stability, or releasing ability of the drug. It is well known that by increasing the proportion of cholesterol located in the lipid bilayers, the particle size [68,69] and the vesicle rigidity also increase [69,70]. Considering that one of the main reasons why lipid vesicles improve drug absorption through the skin is their ability to deform and penetrate between the cells of the stratum corneum, we sought to produce flexible vesicles of the smallest possible size [71,72]. For this, a low ratio of cholesterol to phospholipid (molar ratio 1:17) was chosen for conventional liposomes, since it provides small and ultraflexible vesicles with enough stability. Besides, 85:15% *w/w* lipid:surfactant ratio was used since Ahad et al. demonstrated that it is the most suitable for transdermal delivery of eprosartan mesylate in transferosomes based on P90G:Tween 80 [43]. Additionally, different proportions of ethanol have been studied for preparing ethosomes (20–50% *w/w*). In this case, intermediate concentration (30% *w/w*) was used, considering that it is the most promising ratio for transdermal absorption purposes, according to the characterization results [73].

The initial results (day 0) of size, PDI, zeta potential, drug loading, and phospholipid content are reported in Table 2.

In accordance with other works, size measurements were in the expected range for these types of lipid vesicles. Wu et al. obtained conventional liposomes in a range of 236–374 nm, depending on the percentage of cholesterol included in the formulation [74]. The transferosomal and ethosomal particle dimensions obtained were smaller than those of conventional liposomes, because the presence of the edge-activating substances in the lipid bilayer of the vesicles causes a reduction in the surface tension [75]. Transferosome sizes obtained were similar to the values previously reported by Carreras et al. [75], and ethosomal batches reproduced at exactly the size described by Touitou et al., the original research for this vesicle type (30% *w/w* ethanol content) [44].

PDI values as a measure of the variability in size of the particle populations were always below 0.3, which is the limit value to consider homogeneous populations for lipid-based carriers in drug delivery applications [76,77].

Zeta-potential values of all formulations were negative, as expected, due to the negative charge of the phospholipids. Liposomes showed the strongest charge, probably due to the bigger size and higher phospholipid amount. It has also been reported that the surface charge decreases when increasing the level of cholesterol in a phospholipid membrane [78]. Consequently, our prototypes presented lower values in comparison with other reports where lipid vesicles contained cholesterol in higher proportion [75]. Regarding the transferosomes and ethosomes, they presented a size reduction (probably because of an edge-activator), which implies a lower concentration in phospholipids, thus reducing the negative charge. These results are consistent with the ranges described by Ahad et al. and Touitou et al., who reported values from −5.91 to −14.0 and 4.6 to −4.3 mV for the same type of transferosomes and ethosomes, respectively [43,44].

**Figure 1:**
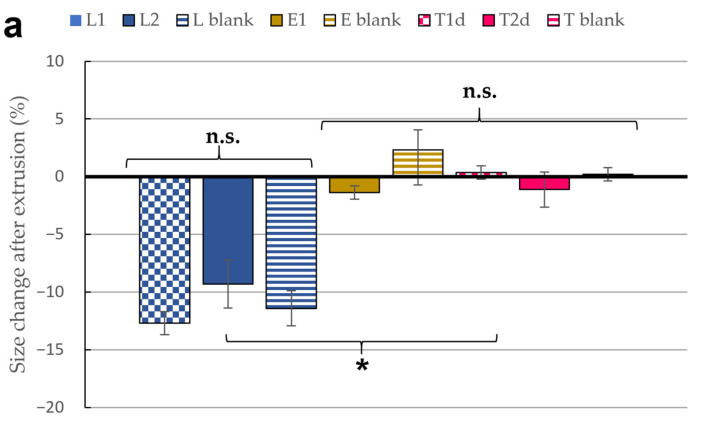

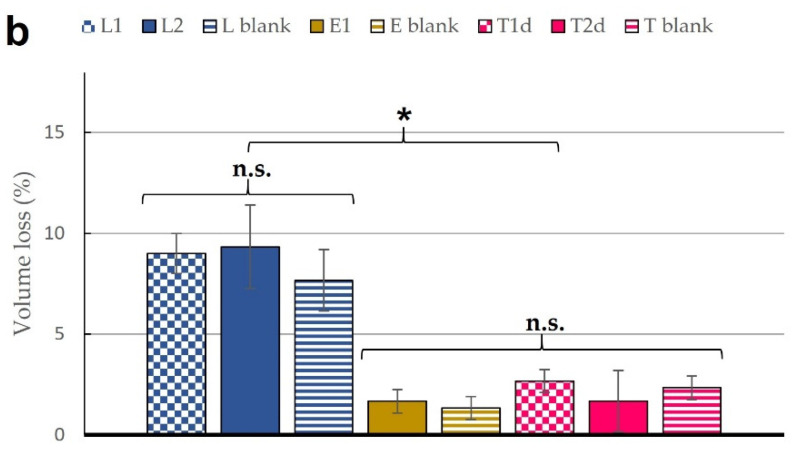
(**a**) Size change after extrusion through 100 nm membrane; (**b**) Volume loss after extrusion through 100 nm membrane (9 passages). All results are expressed as mean ± SD (*n* = 3). * means statistically significant differences (*p* < 0.05), n.s. means no statistically significant differences (*p* > 0.05) using one-way ANOVA followed by Tukey’s multiple comparison test.

Following the work of Jain et al., we used the vesicle size reduction rate and volume loss after cold extrusion as an indirect measurement of the vesicles deformability capacity [54]. Results are presented in Figure 1a,b. Significant differences were obtained between liposomes and ultraflexible vesicles, as expected [79,80]. Liposomes were retained in the 100 nm filters and forced to split into smaller particles to pass the pores. On the contrary, transferosomes and ethosomes—whose initial size was higher than 100 nm—maintained their size during the passage thanks to their flexibility. Likewise, the final volume collected after cold extrusion was inversely related to the vesicle flexibility. As such, no differences were observed in transferosomal or ethosomal batches, while all liposomal prototypes presented a significant reduction compared to their initial volumes. Thus, both techniques confirm the flexibility of transferosomes and ethosomes.

**Figure 2:**
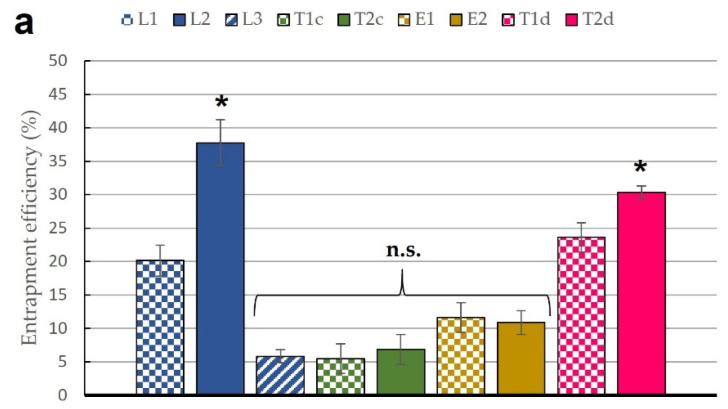

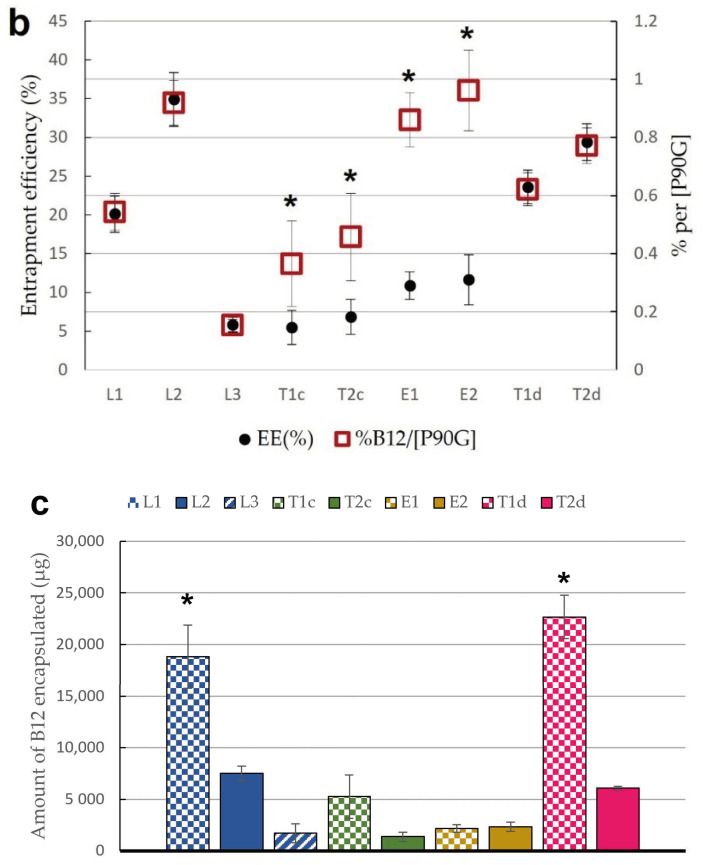
(**a**) Entrapment efficiency (EE) (expressed as a percentage) of the nine prototypes of liposomes, transferosomes, and ethosomes; (**b**) Specific entrapment efficiency rate of the nine prototypes of liposomes, transferosomes, and ethosomes; (**c**) Total amount of B12 encapsulated in 10 mL of liposomal suspension (µg). All results are expressed as mean ± SD (*n* = 3). * means statistically significant differences (*p* < 0.05), n.s. means no statistically significant differences (*p* > 0.05) using one-way ANOVA followed by Tukey’s multiple comparison test.

The entrapment efficiency percentage and total amount of encapsulated B12 are also graphically presented in Figure 2a,c. Hydrophilic compounds often show lower entrapment rates than lipophilic ones. This happens because the entrapment is more efficient if the compound is retained in the phospholipid bilayers, which depends on its primary affinity [48,81]. Arsalan et al. reported a maximum entrapment efficacy of 40% in B12 liposomes when maximizing the amount of lipid included during the vesicle formulation [40]. This result is clearly superior to our L1 data (Figure 2a). To overcome this issue and considering the ability of B12 to solve in aqueous and organic solvents such as methanol, we explored the option of formulating B12 in the organic phase to check any possible difference. In our case, the L2 formulation showed the maximum efficiency in the entrapment process compared to our other prototypes, being significantly different from the other formulations. The comparison between L2 and L1 analyzes the effect of including the B12 in every phase, and the result was higher when the addition was in the lipid one. We also tested the possible influence of differences in the initial dose by preparing L3 formulation. The poor entrapment efficiency in L3 formulation seems to indicate a destabilization of the vesicles with such strategy. T1d and T2d presented an entrapment value within the expected range. At this point, the differences between both transferosomes and L2 could be attributed either to the differences in size, which allowed the incorporation of a higher amount of B12 in the vesicle core, or to the purification method used to remove the non-entrapped drug. While liposomal formulations were centrifuged, T1d and T2d were dialyzed over 24 h [47]. This latter reason is also checked through the EE% and PC% results of T1c and T2c. Both values were lower than expected, probably because of the unsuitability of the centrifugation method for transferosomes. In fact, the supernatant obtained after centrifugation was not completely clear, as it was in the conventional liposomes, and contained still an important amount of liposomes, as demonstrated by a poor phospholipid recovery. The specific encapsulation efficiency parameter was calculated by dividing EE% by PC% [82] (Figure 2b). We could assume that the centrifugation method is adequate for liposomes, as this method is usually successfully used in our laboratories, and the expected amount of phospholipid was recovered after the process. Based on our results, we could conclude that centrifugation was only suitable for liposomal formulations. Ethosomes were not dialyzed because the dialysis process could extract the ethanol from the vesicles [75]. The recovered PC% in ethosomes was around 60% of the initial amount, and the EE% was reasonable for a hydrophilic molecule. In the other samples, phosphatidylcholine was not incorporated in a complete manner, presumably due to material loss during the manufacturing and extrusion processes (Table 2).

However, it should be considered that the objective of this project was to obtain flexible lipid vesicles with the highest possible B12 dose, as saturated systems provide highest skin permeability rates [83]. Figure 2c represents the final amount of B12 per 10 mL of liposomal suspension. Here, we observed that, regardless the encapsulation efficiency values obtained, the highest B12 load was obtained when B12 was introduced in the aqueous phase in a saturated PBS solution (L1 and T1d) (Figure 2c). The higher water volume entrapped by the liposome core contains higher total amounts of B12 compared to the incorporation of the drug in the thin film layer.

**Figure 3:**
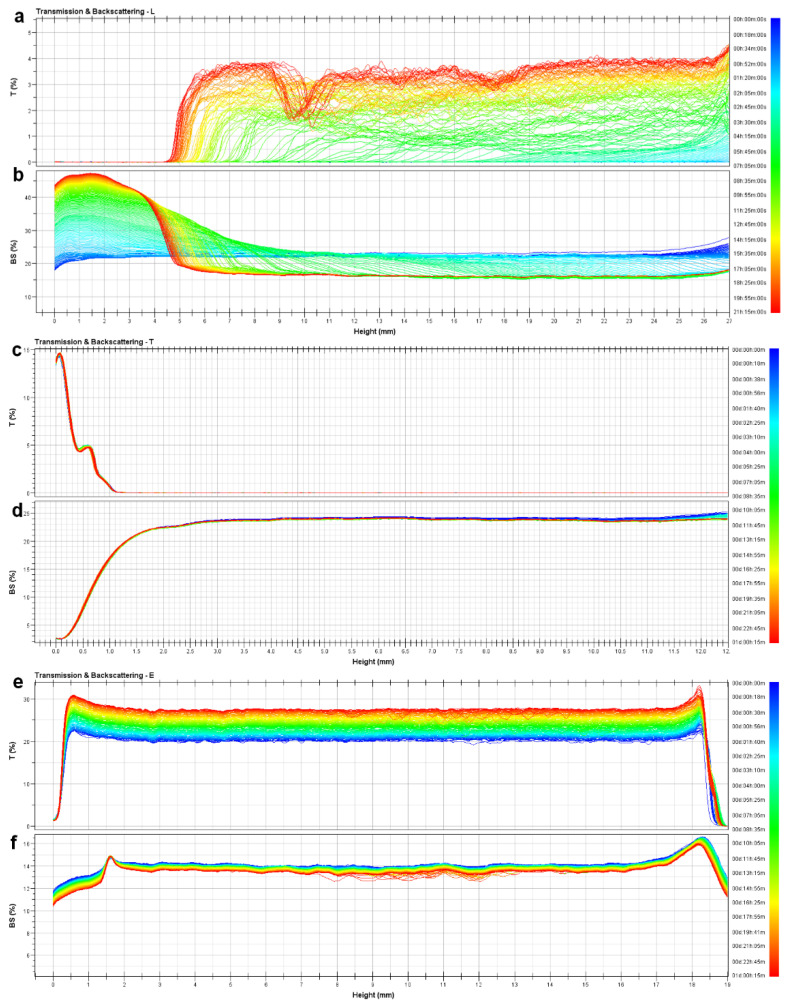
Short-term stability data: (**a**) L1 transmission data; (**b**) L1 backscattering data; (**c**) T1d transmission data; (**d**) T1d backscattering data; (**e**) E1 transmission data; (**f**) E1 backscattering data.

**Figure 4:**
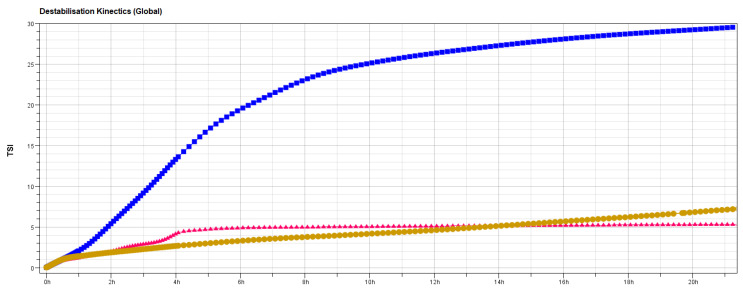
Short-term stability data; L1 TSI (blue), E1 TSI (yellow), and T1d TSI (red).

The short-term stability behavior of the formulations was studied by the Turbiscan Stability Index (TSI), a parameter offered by Turbiscan^TM^ LAB Stability Analyzer device. TSI allows the comparison of samples that present different progression phenomena [84]. Transferosomes were found to be the most stable formulation since no changes in light transmission and backscattering lines were reported over 24 h (Figure 3c,d). A flocculation phenomenon was observed for the ethosomal samples through the backscattering graph (Figure 3f). Its evolution over the whole height of the sample proves a global increase of the particles size (Figure 3e,f). Flocculates could be easily redispersed by gentle shaking. Other aggregative processes, such as coalescence, were discarded, given that no significant long-term size changes were observed after periodically redispersing the samples. On the contrary, liposomal vesicles experienced first a sedimentation process, as the backscattering increases at the bottom of the sample, due to an increase of the concentration in the dispersed phase (sediment) and a decrease at the top of the sample, due to a reduction of the concentration (clarified layer) (Figure 3a,b). Additionally, the smallest particles that remained at the clarified phase showed a tiny flocculation at the end of the investigated times (yellow-red frame). Transmission and backscattering profiles in the work of Cristiano et al. were similar and showed comparable phenomena of flocculation for ethosomes and stability for transferosomes [85]. Therefore, the global destabilization kinetics (Figure 4) confirmed the stability indexes about B12 vesicles (liposome < ethosome < transferosome).

### 3.2. Stability Studies

**Figure 5:**
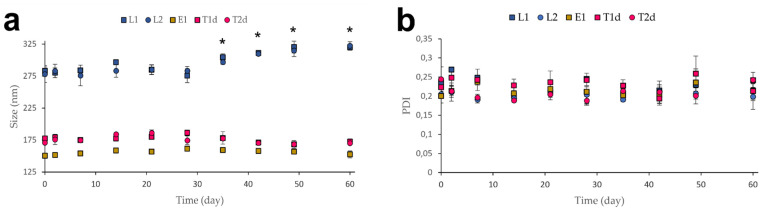
(**a**) Vesicle size over 2 months of storage (4 °C); (**b**) PDI over 2 months of storage (4 °C). All results are expressed as mean ± SD (*n* = 3). * means statistically significant differences when compared to the initial values (*p* < 0.05) using one-way ANOVA followed by Tukey’s multiple comparison test.

Physical and chemical long-term stability was monitored by measuring the vesicle size, PDI, and EE% every week for 2 months and the drug content for 3 months. Figure 5a shows that no significant differences in size as a function of storage time at 4 °C, except for the liposomal formulations after 4 weeks of storage. This fact has been previously reported by other authors [86,87]. On the other hand, intra-type significant changes in PDI were not observed, pointing out a homogeneous evolution in size of vesicles populations (Figure 5b).

**Figure 6:**
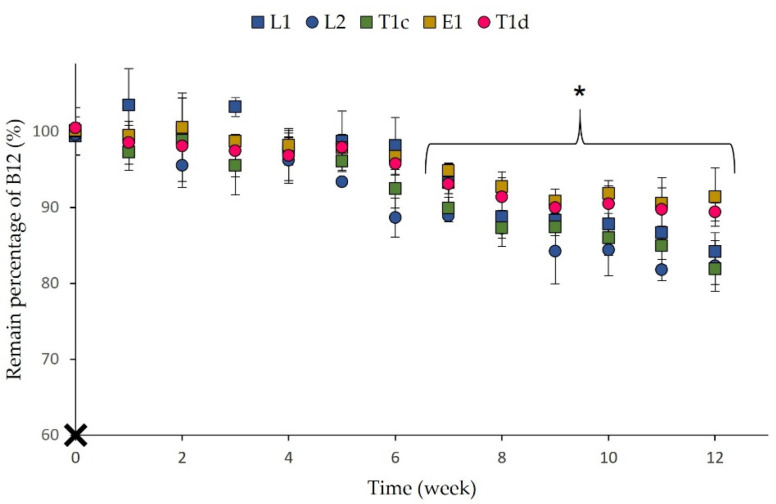
Remaining percentage of B12 amounts during 2 months of storage (4 °C). All results are expressed as mean ± SD (*n* = 3). * means statistically significant differences when compared to initial values (*p* < 0.05) using one-way ANOVA followed by Tukey’s multiple comparison test.

Significant changes in drug content related to the initial amount were detected in all prototypes between weeks 7 and 8 (Figure 6). Recent works have also reported a drug leakage and an effective content loss, especially for liposomes containing hydrophilic drugs, thus supporting our observations [87,88]. Considering these findings, B12 lipid vesicles seemed to exhibit short- and medium-term stability (at least 1 month for all the parameters checked), which makes them suitable for clinical applications. However, to increase the stability for longer-term use, liposome lyophilization could be investigated in the future for these B12 lipid vesicles, since it has been shown as an excellent method for ensuring liposome long-term stability [89,90].

### 3.3. In Vitro Drug Release Studies

**Figure 7:**
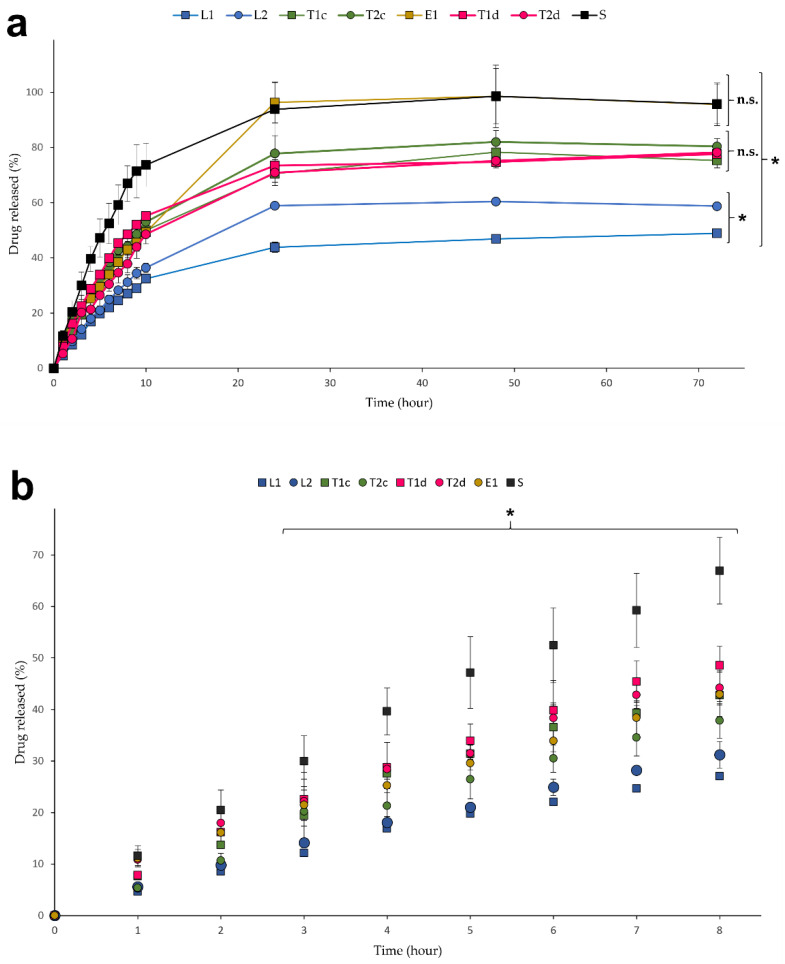
Release profiles from liposomes, transferosomes, ethosomes, and solution after 72 h (**a**) and 8 h (**b**). All results are expressed as mean ± SD (*n* = 6). * means statistically significant differences (*p* < 0.05), n.s. means no statistically significant differences (*p* > 0.05) using two-way ANOVA followed by Bonferroni’s multiple comparison test.

In vitro drug release studies are regularly used in the optimization process of pharmaceutical forms [91]. In this work, the cumulative drug release profiles of the preselected optimized formulations were estimated by a dialysis method using the Franz diffusion cell set-up [92] and presented in Figure 7 and Tables S1 and S2 (Supplementary Materials section). Dialysis methods are appropriate and well accepted to study drug release profiles. Two processes are involved in drug transfer from the donor to the receiver chamber: drug release from the drug reservoir and molecule diffusion through the dialysis membrane, K’ (diffusion rate) and K (release rate) being their respective experimental constants. Using the fitting models, in which K > K’ for first orders transports and K ≈ K’ for zero order transport, we observed that K was higher than K’. Therefore, the limiting step in all cases presented is the drug release from the drug carrier [74].

The B12 solution (S) was used as a control as it represents the drug diffusion profile without limitations. The rest of the lipid vesicle formulations showed a controlled release of drug, as shown in Figure 7b. B12 was released faster during the initial hours from all the tested formulations due to the concentration gradient established between the donor and the receiver media [93]. After 3 h, the B12 amount detected in the receptor medium was significantly higher for the solution than the liposomes, transferosomes, and ethosomes, as expected [94]. Moreover, differences between the release from liposomal and ultraflexible vesicles were also observed, being lower from the liposomes, probably due to their different rigidity. It has been reported that vesicles with considerable bilayer rigidity exhibit higher resistance to drug transport through the liposomal bilayer [95]. The long-term percentage of drug released is probably also affected by this vesicle property. The lowest percentage of drug released corresponded to the liposomes and the highest to the ethosomes, which were able to release around 100% of the encapsulated B12. Nevertheless, the final percentages of released B12 between transferosomes and ethosomes were different (Figure 7a) even though no differences were found in vesicle flexibility during the characterization stage (Figure 1a,b). Possible reasons for this are: the difference of entrapped drug amounts (higher in transferosomes than in ethosomes) that led to different gradients and the vesicle structure and components. We found similar results in B12 release from L1 and L2 formulations profiles at the initial stage (<10 h) even though the B12 entrapped amount in L1 was higher than L2 formulation (Figure 2c). Differences are observed when reaching the plateau. However, the B12 cumulative amounts found in the last L1 samples (44, 46, and 48% at 24, 48, and 72 h, respectively) rose, suggesting that the plateau was not reached and differences may be reduced if the release were extended.

### 3.4. Model Fitting: Kinetic Drug Release Mechanisms

The experimental release data were fitted to different kinetic models to better understand the release profiles: Higuchi, Korsmeyer–Peppas, Kim, Peppas–Sahlin, zero order, and first order. This point is highly recommended since mathematical modeling could help to understand the further in vivo performance of the formulations [96]. Fitting parameters of all the models using the first 10 h data and 72 h data are listed in Table S1, Supplementary Materials section. The AIC was used as a comparative of the goodness of fit (also listed in Table S1, Supplementary Materials section). In general, the Korsmeyer–Peppas model presented the lowest AIC values, indicating an accurate fitting, for almost all formulations. However, in certain cases (T1d 10 h, T2c 72 h, E1 72 h and S), the first order was the best model. The Kim model is a modification of the Korsmeyer–Peppas one that considers a possible burst effect. This burst effect (represented by parameter “b”) was neglected by the fitting, and the results of both models match. Burst release effect of drugs is frequently related to “dose dumping”, an event to avoid in a controlled release system that was demonstrated not to happen in our prototypes [97,98].

The main difference between the first order model and the Korsmeyer–Peppas one is the mechanisms underlying the release process. First order only reflects a passive diffusion, while Korsmeyer–Peppas also considers other effects, such as relaxation or matrix erosion. Contrary to what was expected for ultraflexible vesicles, the Korsmeyer–Peppas model was more suitable for most of the cases, pointing out that they present a mixed release mechanism. It seems that relaxation has a stronger influence during the first steps and gets diluted during longer sampling times, in favor of passive diffusion. This behavior can be deduced from the values of the release exponent “n”, which presents intermediate values (0.5–0.85) when the initial 10 h data are fitted and low values (<0.5) when 72 h data are included. Finally, the *R*^2^ values from both models are also listed in Table S2 (Supplementary Materials section). We could observe from their analysis that the models used are suitable for explaining the release process (they account for >90% of the experimental data variation) [57,58].

### 3.5. Tape-Stripping Drug Penetration Studies

The stratum corneum layers depth in porcine skin was determined by infrared densitometry using a standardized procedure [66]. The total thickness obtained was around 20 µm, according with the standard values between 17–28 µm, previously reported by Jacobi et al. [99]. Tape-stripping studies were performed using the most promising formulations according to the afore-presented data: L1, L2, T1d, T2d, and E1. As incubation times, 2 h, 4 h, and 6 h were selected, covering the longest time a topical formulation would be maintained onto the skin. Besides, longer incubation times lead to overhydration of the skin and removal of the whole stratum corneum in a single strip [83]. The B12 solution 0.5% *w/v* (S) was used as a reference since it represents the free diffusion, and no enhancing absorption effects are expected from the vehicle.

**Figure 8:**
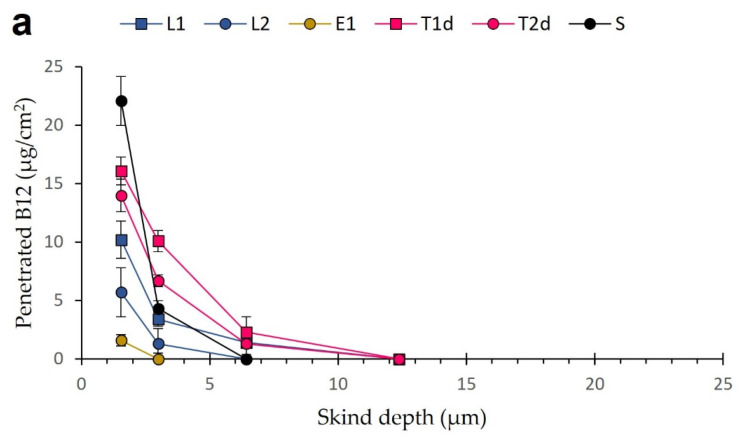

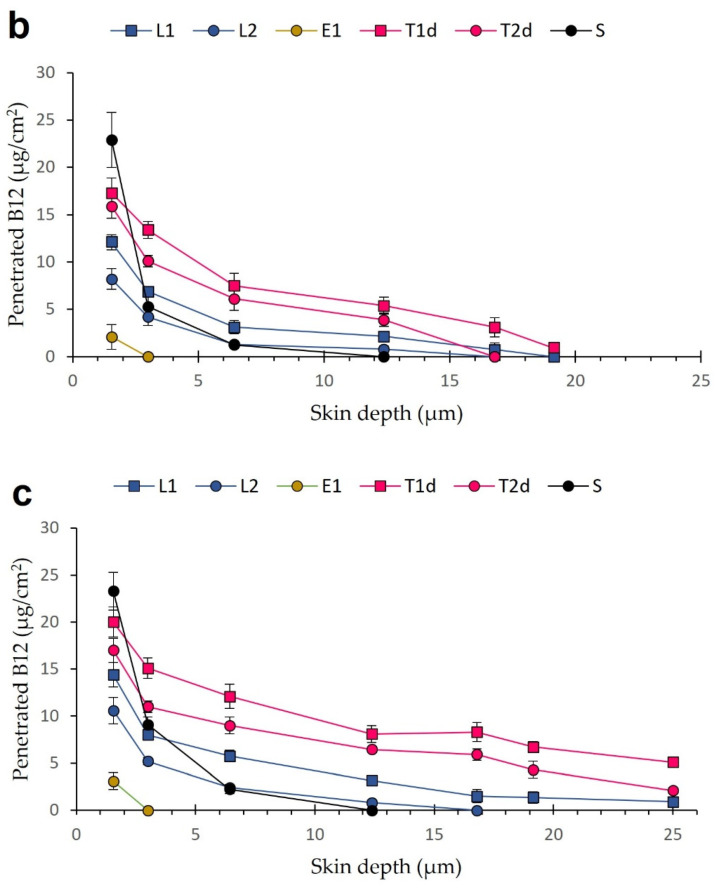
Penetration profiles of B12 delivered from liposomes, transferosomes, ethosomes, and solution after 2 h (**a**), 4 h (**b**), and 6 h (**c**). All results are expressed as mean ± SD (*n* = 3).

Figure 8 shows the progressive B12 penetration in the different layers of the stratum corneum at different incubation periods. In general, the amounts of B12 detected in the deeper layers of the skin increased as a function of the incubation time, as expected. As shown, B12 solution presented the highest amount of B12 extracted in the first strip regardless of the incubation time. Two reasons can explain this fact: immediate availability of B12 on the skin leading to a quick distribution over the first stratum corneum layer, where it is retained; and a deficient cleaning of the skin surface. Concerning the latter, many authors discard this strip because the inaccuracy of the data is typically high and considered as an experimental artifact [100]. However, the rest of B12 from the solution did not penetrate the stratum corneum nor the deeper skin layers, and only low quantifiable concentrations were detected up to the third strip, which corresponds to 6 µm depth, after 6 h. These findings confirm the inability of B12 to diffuse through intact skin. The ethosome samples presented the lowest quantifiable amounts among all the vesicles tested, probably due to the low B12 load and the gradual release. Samples could have easily been under the detection/quantification limits.

The best penetration results were obtained using liposomes and transferosomes. L2 carries less B12 amounts than the other prototypes, consequently showing a considerably lower B12 penetration amounts, only until approximately 15 µm depth (Figure 8c). Nevertheless, L1, T1d, and T2d vesicles allowed the B12 to reach the dermis (>25 µm). L1 and T2d contained similar B12 doses, but after 4 and 6 h of incubation, the transferosomes showed higher permeation rates up to the deepest layers (Figure 8b,c). This means that the stratum corneum is saturated in B12 at that time point, and a diffusion gradient to the deeper layers starts. T1d showed the highest permeation profiles. Our penetration results suggest that transferosome enhancing effect was much higher than the one of liposomes and are in agreement with Abd et al. [101], who compared the penetration of a hydrophilic drug (caffeine) delivered from liposomes, transferosomes, and solution.

B12 has been delivered through the skin using chemical and physical methods by other authors. Recently, Ramöller et al. achieved an effective delivery of B12 to plasma in a rat model. Rapidly dissolving microneedle allowed the permeation of 60% of dose after 2 h post-insertion, achieving 0.4 µg/mL plasma levels [28]. Yang et al. studied in vitro the effects of chemical enhancers and physical methods in topical administration of B12. Chemical enhancers (ethanol, oleic acid, and propylene glycol) allowed an effective permeability of B12 in comparison with passive diffusion. The permeability enhancement offered by iontophoresis was around 2-fold in comparison with a combination of all enhancers (50% ethanol, 10% oleic acid, and 40% propylene glycol) [27]. Although these methods have proven to effectively delivery B12, they present several inconveniences in B12 delivery for atopic dermatitis. For example, they were not able to localize their effects in the epidermis and dermis leading to an absorption of B12 to systemic circulation, which is unnecessary for psoriatic and atopic dermatitis patients. Additionally, local skin reactions and poor effectiveness in combination with hydrophilic molecules has been reported for chemical enhancers [102]. Moreover, ultraflexible lipid vesicles are technically easier to produce and a low cost in comparison to microneedles and iontophoresis devices [102].

From the results presented here, it can be concluded that the developed formulations are able to efficiently deliver B12 to the deeper skin layers after 6 h, as desired. We also demonstrate that B12 does not diffuse through the stratum corneum if formulated in water media. Further studies should be performed to formulate these vesicles in adequate dosage forms for an effective topical application of B12 that can be transferred to the clinic.

## 4. Conclusions

Enhanced penetration of B12 through the skin is possible using the lipid vesicle formulations (L1, T2d, and T1d) designed in this work, which opens the possibility to improve the clinical results obtained in previous studies and to investigate its utility for topical treatment of atopic dermatitis and psoriasis. Several particle properties such as size, stability, and purification method were revealed as key parameters to achieve a suitable and efficient production of lipid vesicles. Skin permeability studies should be further investigated to elucidate if these nanosystems are able not only to increase the penetration in the skin but also to allow the passage of B12 to the systemic circulation, thus broadening the possible applications of the systems for the treatment of other pathologies related with B12 deficiencies.

## Figures and Tables

**Table 1:** Quantitative composition of the different lipid vesicle systems, reconstitution conditions, and purification method.

Components (% *w/v*)	Formulations								
	L1	L2	L3	T1c	T2c	T1d	T2d	E1	E2
**Phospholipon 90G ^®^**	4.5	4.5	4.5	4.5	4.5	4.5	4.5	3	3
**Cholesterol**	0.135	0.135	0.135	-	-	-	-	-	-
**Tween 80**	-	-	-	0.675	0.675	0.675	0.675	-	-
**B12 ^1^**	1 ^1^	0.2 ^2^	0.2 ^1^	1 ^1^	0.2 ^2^	1 ^1^	0.2 ^2^	0.2 ^2^	0.2 ^3^
**Reconstitution solvent (mL)**	10 mL PBS	10 mL PBS	10 mL PBS	10 mL PBS	10 mL PBS	10 mL PBS	10 mL PBS	20 mL Water	20 mL Water
**Purification method ^4^**	C	C	C	C	C	D	D	C	C

^1^ B12 added in reconstitution solution. ^2^ B12 added in the organic-lipid phase. ^3^ B12 added dropwise in solution. ^4^ C: centrifugation; D: dialysis (24 h). PBS: Phosphate Buffer Saline.

**Table 2:** Characterization of the B12 lipid vesicles in terms of size, polydispersity index (PDI), zeta-potential, drug loading, and phospholipid content (PC). All results are expressed as mean ± SD (*n* = 3).

	Formulations								
	L1	L2	L3	T1c	T2c	T1d	T2d	E1	E2
**Size (nm)**	283 ± 6	278 ± 13	275 ± 9	175 ± 5	169 ± 10	177 ± 4	171 ± 3	150 ± 5	141 ± 11
**PDI**	0.269 ± 0.07	0.205 ± 0.002	0.215 ± 0.009	0.239 ± 0.02	0.261 ± 0.03	0.223 ± 0.01	0.244 ± 0.03	0.200 ± 0.003	0.193 ± 0.01
**Zeta potential (mV)**	−11.2 ± 0.12	−10.1 ± 0.3	−9.55 ± 0.84	−4.77 ± 0.13	−5.01 ± 0.21	−5.51 ± 0.17	−5.17 ± 0.37	−4.63 ± 0.75	−5.35 ± 1.58
**Drug loading (mg per mL)**	1.9 ± 0.3	0.75 ± 0.07	0.17 ± 0.09	0.52 ± 0.02	0.14 ± 0.04	2.2 ± 0.2	0.60 ± 0.02	0.22 ± 0.03	0.23 ± 0.04
**PC (%)**	80 ± 4	84 ± 7	77 ± 2	33 ± 3	30 ± 0.9	86 ± 8	82 ± 5	66 ± 2	60 ± 5

## Data Availability

Data is contained within the article.

## Supplementary Materials

The following are available online at https://www.mdpi.com/1999-4923/13/3/418/s1, Figure S1: Release parameters (10 and 72 h) for release kinetic models: Higuchi, Korsmeyer–Peppas, Kim, Peppas–Sahlin, zero order, and first order; Figure S2: *R*^2^ values of Korsmeyer–Peppas and first order models for the B12 vesicles release data (10 and 72 h).
