# Supplementary material for: Cyanocobalamin Ultraflexible Lipid Vesicles: Characterization and In Vitro Evaluation of Drug-Skin Depth Profiles
Source: Pharmaceutics. 2021 Mar 20;13(3):418. doi: 10.3390/pharmaceutics13030418 (PMC8003749; doi:10.3390/pharmaceutics13030418)
Supplement: Supplementary file 1 [file pharmaceutics-13-00418-s001.pdf]

pharmaceutics

MDPI

Supplementary Materials: Cyanocobalamin Ultraflexible Lipid Vesicles: Characterization and In Vitro Evaluation of Drug-Skin Depth Profiles

Antonio José Guillot, Enrique Jornet-Mollá, Natalia Landsberg, Carmen Milián-Guimerá, M. Carmen Montesinos, Teresa M. Garrigues and Ana Melero

Table S1. Release parameters (10 and 72 h) for release kinetic models: Higuchi, Korsmeyer-Peppas, Kim, Peppas-Sahlin, zero orden and first order. All results are expressed as mean ± SD (*n* = 6).

		Higuchi		Korsmeyer-Peppas			Kim				Peppas-Sahlin				Zero order		First order	
Prototype	Time (h)	k	AIC	k	n	AIC	k	n	b	AIC	K_1	K_2	n	AIC	Kd	AIC	Kd	AIC
L1	10	9.12 ± 0.51	44.75	5.41 ± 0.33	0.77 ± 0.02	18.06	5.41 ± 0.33	0.77 ± 0.02	0	18.06	2.89 ± 0.31	2.99 ± 0.45	0.45 ± 0.02	22.84	3.46 ± 0.06	37.44	0.04 ± 0.001	26.3
	72	7.32 ± 0.13	80.9	11.31 ± 0.34	0.36 ± 0.05	76.04	11.31 ± 0.34	0.36 ± 0.05	0	76.04	3.78 ± 0.32	4.34 ± 0.44	0.25 ± 0.007	90.91	0.96 ± 0.86	107.29	0.02 ± 0.001	108.83
L2	10	10.36 ± 0.63	52.77	5.85 ± 0.65	0.80 ± 0.03	48.21	5.85 ± 0.65	0.80 ± 0.03	0	48.21	2.68 ± 0.15	3.49 ± 0.58	0.46 ± 0.02	14.33	3.9 ± 0.02	36.38	0.04 ± 0.008	17.75
	72	8.92 ± 0.35	95.73	12.59 ± 0.72	0.39 ± 0.01	87.80	12.59 ± 0.72	0.39 ± 0.01	0	87.80	5.71 ± 1.07	7.45 ± 1.58	0.23 ± 0.24	87.81	1.19 ± 0.04	111.64	0.03 ± 0.002	99.58
T1c	10	14.51 ± 0.65	48.21	9.07 ± 2.40	0.75 ± 0.08	31.89	9.07 ± 2.40	0.75 ± 0.08	0	31.89	0	9.07 ± 2.13	0.37 ± 0.03	33.89	5.49 ± 0.29	49.6	0.07 ± 0.004	34.32
	72	11.68 ± 0.36	87.80	18.18 ± 1.08	0.36 ± 0.01	89.00	18.18 ± 1.08	0.36 ± 0.01	0	89.00	7.78 ± 0.26	11.05 ± 0.85	0.21 ± 0.007	91.85	1.53 ± 0.07	119.71	0.06 ± 0.005	92.05
T2c	10	11.30 ± 0.76	47.88	10.87 ± 0.54	0.68 ± 0.02	24.95	10.87 ± 0.54	0.68 ± 0.02	0	24.95	6.15 ± 1.45	5.26 ± 1.17	0.42 ± 0.03	27.67	5.80 ± 0.31	54.18	0.07 ± 0.005	39.01
	72	13.03 ± 0.59	97.24	19.75 ± 1.29	0.36 ± 0.01	89.30	19.75 ± 1.29	0.36 ± 0.01	0	89.30	7.92 ± 0.04	12.48 ± 0.64	0.2 ± 0.006	92.08	1.63 ± 0.09	121.45	0.07 ± 0.007	86.75
T1d	10	16.7 ± 1.56	55.28	9.90 ± 1.63	0.76 ± 0.04	30.15	9.90 ± 1.63	0.76 ± 0.04	0	30.15	0	9.9 ± 1.5	0.38 ± 0.02	32.15	6.12 ± 0.58	50.43	0.08 ± 0.01	18.2
	72	12.19 ± 0.97	100.9	21.07 ± 2.50	0.33 ± 0.02	91.98	21.07 ± 2.50	0.33 ± 0.02	0	91.98	8.97 ± 0.86	12.72 ± 1.72	0.19 ± 0.01	34.69	1.57 ± 0.12	122.57	0.07 ± 0.01	93.82
T2d	10	11.30 ± 0.87	56.56	6.57 ± 1.26	0.86 ± 0.11	32.51	6.57 ± 1.26	0.86 ± 0.11	0	32.51	3.42 ± 1.86	3.64 ± 2.2	0.50 ± 0.07	35.20	4.97 ± 0.35	38.80	0.06 ± 0.005	32.76
	72	13.03 ± 0.45	91.28	15.23 ± 1.23	0.41 ± 0.01	89.04	15.23 ± 1.23	0.41 ± 0.01	0	89.04	6.56 ± 0.41	9.34 ± 0.88	0.23 ± 0.008	91.93	1.52 ± 0.05	116.72	0.05 ± 0.006	89.29
E1	10	14.31 ± 1.3	46.54	9.93 ± 1.27	0.69 ± 0.03	17.21	9.93 ± 1.27	0.69 ± 0.03	0	17.21	5.63 ± 0.52	4.77 ± 3.45	0.48 ± 0.09	14.33	5.39 ± 0.05	14.33	0.07 ± 0.009	38.46
	72	13.83 ± 1.08	96.13	16.75 ± 2.50	0.44 ± 0.03	96.59	16.75 ± 2.50	0.44 ± 0.03	0	96.59	6.6 ± 0.91	10.93 ± 1.60	0.24 ± 0.02	99.22	1.89 ± 0.01	99.22	0.07 ± 0.01	73.66
S	10	21.8 ± 1.5	61.35	13.35 ± 2.65	0.76 ± 0.05	37.41	13.35 ± 2.65	0.76 ± 0.05	0	37.41	0	13.33 ± 2.65	0.38 ± 0.02	39.41	8.26 ± 0.09	56.48	0.13 ± 0.02	35.17
	72	15.85 ± 2.57	110.5	29.22 ± 4.55	0.31 ± 0.02	100.6	29.22 ± 4.55	0.31 ± 0.02	0	100.6	17.14 ± 2.29	3.3 ± 0.83	0.43 ± 0.02	97.46	2.02 ± 0.02	130.60	0.12 ± 0.02	52.81

Table S2. R^2 values of Korsmeyer-Peppas and first order models for the B12 vesicles release data (10 and 72 h) (*n* = 6).

	R^2															
Prototype	L1		L2		T1c		T2c		T1d		T2d		E1		S	
Time (h)	10	24	10	24	10	24	10	24	10	24	10	24	10	24	10	24
Korsmeyer-Peppas	0.996	0.916	0.999	0.899	0.994	0.911	0.997	0.917	0.996	0.890	0.993	0.916	0.998	0.908	0.996	0.868
First order	0.995	0.879	0.998	0.884	0.994	0.891	0.994	0.915	0.997	0.881	0.992	0.915	0.994	0.917	0.997	0.935
